# Diagnostic dilemma in a case of malignant mixed mullerian tumor of the cervix

**DOI:** 10.1186/1477-7819-4-36

**Published:** 2006-07-01

**Authors:** Amita Maheshwari, Sudeep Gupta, Tanuja Shet, Rekha Wuntkal, Hemant B Tongaonkar

**Affiliations:** 1 Gynaecologic Oncology Service, Department of Surgical Oncology, Tata Memorial Hospital, Parel, Mumbai, India; 2 Department of Medical Oncology, Tata Memorial Hospital, Parel, Mumbai, India

## Abstract

**Background:**

Malignant mixed mullerian tumors (MMMT) are rare biphasic malignant neoplasm. The commonest site of their occurrence in female genital tract is body of the uterus. MMMT of the cervix is extremely rare.

**Case presentation:**

We report the clinical, pathological and immunohistochemical profile and diagnostic difficulties in a case of giant MMMT of the cervix in a postmenopausal woman who presented with a large cervical mass. On microscopic examination, initially tumor appeared to be endometrial stromal sarcoma, however, immunohistochemical examination revealed the biphasic nature of the tumor. The malignant epithelial component was basaloid squamous carcinoma with homologous sarcomatous component. The patient was treated with surgery. However, she experienced vaginal vault recurrence four months after the initial treatment, which was successfully treated with pelvic radiotherapy.

**Conclusion:**

Accurate diagnosis of cervical MMMT is important for appropriate treatment of the patient.

## Background

Malignant mixed mullerian tumors (MMMT) are rare biphasic malignant neoplasm. The commonest site of occurrence of female genital tract MMMT is the uterine corpus. MMMT of the cervix is extremely rare. It was first described by Ferriera in 1951 [1]. Approximately 50 cases have been reported in the English language literature on this rare entity [2].

We report the clinical, pathological and immunohistochemical profile and diagnostic difficulties in a case of giant MMMT of the cervix where malignant epithelial component resembled endometrial stromal sarcoma.

## Case presentation

A 60-year-old postmenopausal woman presented with lump in lower abdomen of 2 months duration. Abdominal examination revealed a large, irregular, firm supra-pubic mass. On pelvic examination, the same mass was felt in the vagina. Cervix and uterus were not felt separately from the mass. Computed tomography (CT) scan showed a large, well defined heterogeneous abdomino-pelvic mass. Serum CA-125 level was 52.3 U/mL. Exploratory laparotomy was performed, which revealed a large, bosselated mass arising from the cervix. Body of the uterus, bilateral ovaries, tubes and other abdomino-pelvic organs were within normal limits. Total abdominal hysterectomy with bilateral salpingo-oophorectomy with complete excision of the mass was done after identifying the ureters from pelvic brim to their entry into the urinary bladder. Her postoperative recovery was uneventful.

The patient was kept under close observation after initial treatment. Four months after surgery, on routine follow-up, vaginal cytology showed the malignant squamous component of MMMT. The recurrence was confirmed by colposcopy-guided biopsy from the vaginal vault. Physical examination and CT-scan did not reveal any evidence of disease elsewhere. This recurrence was treated with radical pelvic radiotherapy (RT) (external pelvic RT 60 Gy in 30 fractions plus 2 intracavitary applications of 6 Gy each). The patient is free of disease at eighteen months after the treatment of recurrence.

### Pathologic findings

Gross examination of the surgical specimen revealed a 28 × 20 × 15 cm bosselated, mass arising from the cervix. The cervical canal was distorted because of the mass. On cut surface, the mass was fleshy, solid with cystic areas.

**Figure 1:**
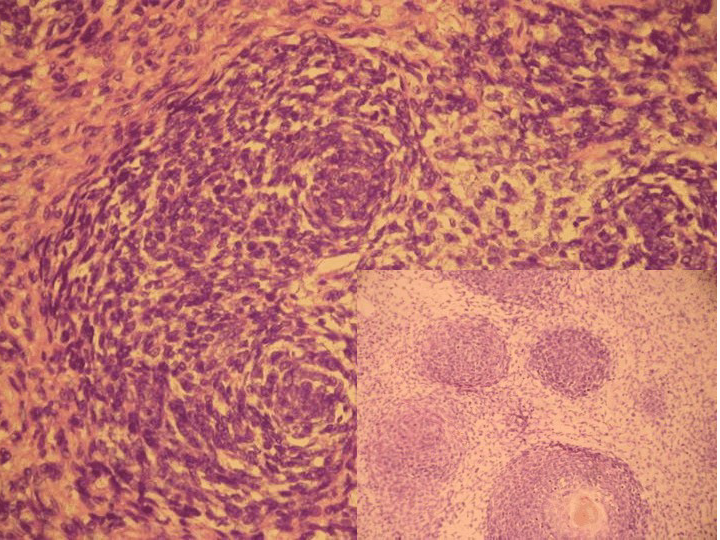
Photomicrograph showing a basaloid tumor with small cells arranged in whorled manner (H&E ×200). Inset: Squamous differentiation in center of the whorls (H&E ×100).

**Figure 2:**
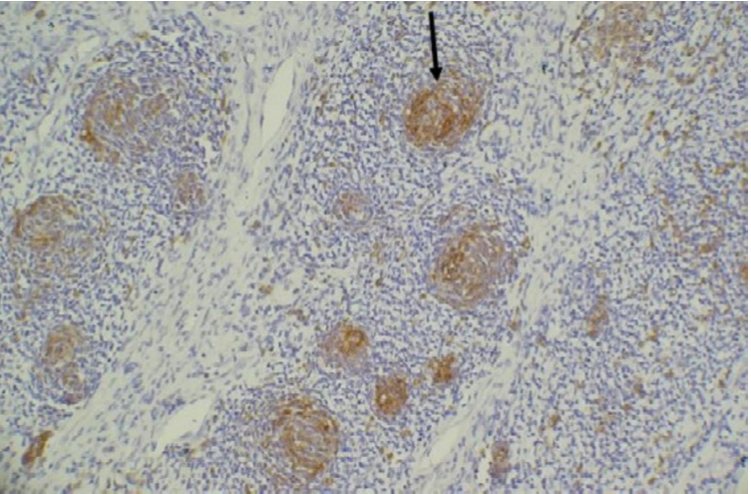
On immunohistochemistry the whorled areas were cytokeratin positive while the surrounding sarcomatous element was negative (ABC ×100).

**Figure 3:**
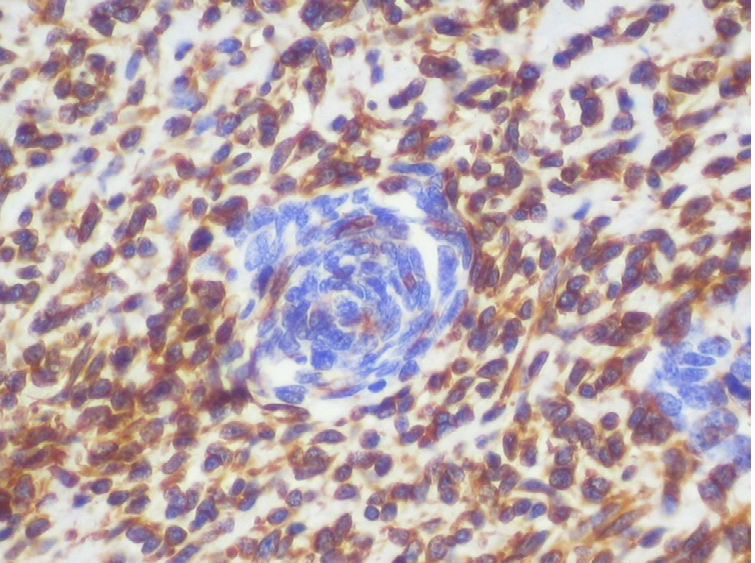
The biphasic nature of the tumor was further highlighted on the vimentin stain., whereby only the stromal element showed diffuse vimentin positivity (ABC ×100).

On hematomylin and eosin staining, the entire tumor showed cellular whorls dispersed amidst pale sarcomatous stroma. The cells in whorls were round to short spindle shaped and had moderate mitotic activity (figure 1). Although no vessels were seen in the center of these whorls, the whorled appearance was reminiscent of an endometrial stromal sarcoma. Hence, the preliminary diagnosis of endometrial stromal sarcoma (ESS) was made. Additional sections however revealed that whorled areas had basaloid squamous carcinoma in the center (figure 1 inset). A panel of immunohistochemical tests was performed and whorled basaloid areas were positive for cytokeratin (figure 2), epithelial membrane antigen and CD 10 but were negative for vimentin confirming their epithelial nature. The stromal component of the tumor was highlighted on vimentin stain (figure 3). The stromal element was sarcomatous and showed diffuse vimentin and focal SMA positivity. SMA, S-100, calponin, inhibin, Mic-2, desmin and myoglobin were negative in the basaloid islands. No heterologous elements were seen. The final diagnosis was homologous malignant mixed mullerian tumor with basaloid squamous carcinoma.

## Discussion

Cervical MMMT usually occur in post-menopausal women with mean age at diagnosis of 61 to 69 years although the age range in the literature varies from 12 to 93 years [3-6]. The commonest clinical features are vaginal bleeding, abnormal vaginal cytology and polypoidal cervical mass [3-6]. However, the presenting feature in our case was a large abdomino-pelvic mass.

MMMT are biphasic tumors that consist of an admixture of malignant epithelial and mesenchymal components. The epithelial component represents a variety of different histological sub-types, alone or in combination, which include squamous cell carcinoma, basaloid squamous carcinoma, adenocarcinoma, adeno-squamous carcinoma, adenoid-basal carcinoma, adenoid-cystic carcinoma and undifferentiated carcinoma [3-5]. The sarcomatous component may be homologous (fibroblasts and smooth muscle) or heterologous (cartilage, striated muscle, bone etc). On immunohistochemical examination, both epithelial and sarcomatous components of MMMT may show positivity for broad spectrum cytokeratins, high molecular weight cytokeratin, low molecular weight cytokeratin and epithelial membrane antigen. Sarcomatous components may be positive for vimentin, desmin, muscle specific actin (MSA) and smooth muscle-specific actin (SMA) [3].

These tumors must be differentiated from other tumors on histopathology. Our case illustrates the varied pattern of MMMT and its tendency to mimic ESS. The islands of squamous differentiation were the clue to the diagnosis, as they are not seen in ESS. Extrauterine ESS arising in the cervix is extremely rare with only three reported cases available in the English literature [2]. These tumors usually arise in the endocervical polyp and histologically resemble ESS of the uterus. ESS are classically positive for CD10 which overlaps with CD 10 positivity in some cases of MMMT [7] as seen in our case. Other tumors that may mimic MMMT and should be differentiated from it are sarcomatoid carcinoma and mullerian adenosarcoma. In sarcomatoid carcinoma, the carcinomatous component frequently merges with the sarcomatous, as opposed to the sharp distinction seen in MMMT. Mullerian adenosarcoma is relatively easy to differentiate from MMMT, as the epithelial component in the former is benign.

Grayson *et al*., [3] studied human papillomavirus (HPV) status in eight patients with cervical MMMT. In all cases, HPV-DNA was detected by polymerase chain reaction. Interestingly, using in-situ hybridization technique they demonstrated HPV-16 DNA in the nuclei of both epithelial and sarcomatous components in three cases. This observation supports a metaplastic theory of histogenesis of these tumors.

Clement *et al*., [5] reported the largest single series of nine cases of cervical MMMT. They suggested that compared to its uterine counterpart, cervical MMMT is more often confined to the uterus at presentation, frequently has non-glandular epithelial component and may have better prognosis. Due to rarity of this tumor, no evidence based management guidelines are available. Surgery is the principal modality of treatment. Although adjuvant chemotherapy and/or radiotherapy have been used, their role is not well-defined. Radical radiotherapy with or without chemotherapy is recommended for locally advanced disease. Patients with metastatic disease are treated with palliative chemotherapy. Commonly used chemotherapeutic agents are cisplatin, doxorubicin, ifosphamide or cyclophosphamide. The clinical behavior of these tumors is dominated by the carcinomatous component [8]. In our case, only the epithelial squamous component was seen at recurrence, which was successfully treated by radiotherapy that is also the treatment of choice for cervical squamous cell carcinoma.

## Conclusion

Cervical malignant mixed mullerian tumors are rare malignancies that may at times present diagnostic difficulties to the clinician. They are best treated by surgery with or without adjuvant radiation and/or chemotherapy. Accurate diagnosis is essential for appropriate treatment and prognosticating the disease.

## Competing interests

The author(s) declare that they have no competing interests.

## Authors' contributions

**AM **conceived the idea and drafted the manuscript, **SG **participated in the patients management and help in preperation of the manuscript. **TS, RW **did the literature search and helped in drafting the manuscript. **HG **did the overall supervision, and edited the final manuscript. All authors read and approved the final manuscript.

## References

[B1] Ferriera HP (1951). A case of mixed mesodermal tumor of the uterine cervix. J Obstet Gynaecol Br Emp.

[B2] Wright JD, Rosenblum K, Huettner PC, Mutch DG, Rader JS, Powell MA, Gibb RK (2005). Cervical sarcomas: An analysis of incidence and outcome. Gynecol Oncol.

[B3] Grayson W, Taylor LF, Cooper K (2001). Carcinosarcoma of uterine cervix: a report of eight cases with immunohistochemical analysis and evaluation of human papillomavirus status. Am J Surg Pathol.

[B4] Abell MR, Ramirez JA (1973). Sarcomas and Carcinosarcomas of the uterine cervix. Cancer.

[B5] Clement PB, Zubovits JT, Young RH, Scully RE (1998). Malignant mullerian mixed tumors of the uterine cervix: a report of nine cases of a neoplasm with morphology often different from its counterpart in the corpus. Int J Gynecol Pathol.

[B6] Sharma NK, Sorosky JI, Bender D, Fletcher MS, Sood AK (2005). Malignant mixed mullerian tumor (MMMT) of the cervix. Gynecol Oncol.

[B7] Mikami Y, Hata S, Kivokawa T, Manabe T (2002). Expression of CD10 in malignant mullerian mixed tumors and adenosarcoma: an immunohistochemical study. Mod Pathol.

[B8] Costa MJ, Guinee D (2000). CD34 immunohistochemistry in female genital tract carcinosarcoma (malignant mixed mullerian tumors) supports a dominant role of the carcinomatous component. Appl Immunohistochem Mol Morphol.

